# Spatio-Temporal Regulation of RhoGTPases Signaling by Myosin II

**DOI:** 10.3389/fcell.2019.00090

**Published:** 2019-05-28

**Authors:** Selwin K. Wu, Rashmi Priya

**Affiliations:** ^1^Department of Cell Biology, Harvard Medical School, Boston, MA, United States; ^2^Department of Pediatric Oncology, Dana-Farber Cancer Institute, Boston, MA, United States; ^3^Department of Developmental Genetics, Max Planck Institute for Heart and Lung Research, Bad Nauheim, Germany

**Keywords:** pulsatility, morphogenesis, GTPase signaling, adhesion and migration, active fluid media, actomyosin

## Abstract

RhoGTPase activation of non-muscle myosin II regulates cell division, extrusion, adhesion, migration, and tissue morphogenesis. However, the regulation of myosin II and mechanotransduction is not straightforward. Increasingly, the role of myosin II on the feedback regulation of RhoGTPase signaling is emerging. Indeed, myosin II controls RhoGTPase signaling through multiple mechanisms, namely contractility driven advection, scaffolding, and sequestration of signaling molecules. Here we discuss these mechanisms by which myosin II regulates RhoGTPase signaling in cell adhesion, migration, and tissue morphogenesis.

## Introduction

Non-muscle myosin II is a major determinant of cell and tissue morphogenesis (Vicente-Manzanares et al., 2009; Wu and Yap, 2013; Priya and Yap, 2015). Myosin II is best characterized as a cytoskeletal motor-protein, which binds to filamentous actin and generates forces as an actomyosin complex (Even-Ram et al., 2007; Conti and Adelstein, 2008; Vicente-Manzanares et al., 2009; Lee et al., 2010; Gomez et al., 2011; Kuo et al., 2011; Shin et al., 2014; Priya et al., 2015). Myosin II regulates forces in cells by cross-linking the filamentous actin network across the cytoplasmic cortex of a cell (Cai et al., 2006, 2010; Luo et al., 2013). In extension to the well-established pathway of active Rho GTPase activating myosin II, strikingly, myosin II can also feedback to regulate Rho GTP signaling by scaffolding signaling molecules and through generating contractile forces (Priya et al., 2015; Munjal et al., 2015).

## Actomyosin Pulsatility Feedbacks to Rho Signaling

**FIGURE 1:**
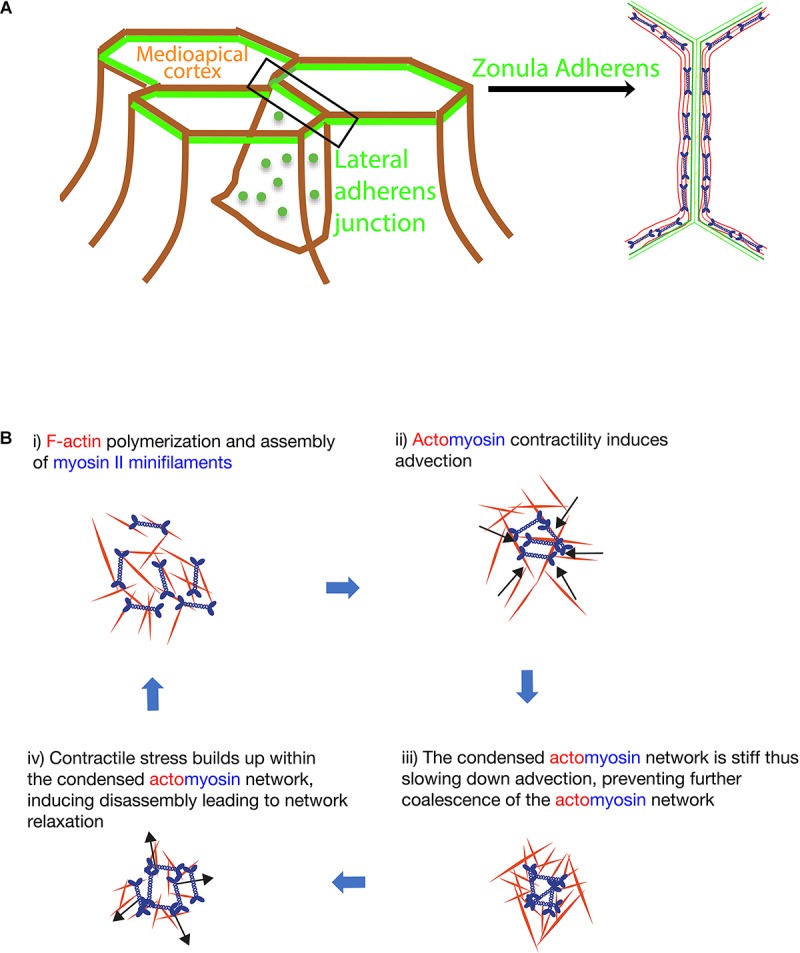
**(A)** Localization of the medial apical cortex, zonula adherens and lateral adherens junction in epithelial tissues (left). A top down view of the organization of actomyosin bundles at the zonula adherens in established epithelial monolayer (right). **(B)** Model for self-organization of the biomechanical cortical network at the lateral adherens junction and the medial apical cortex of cells.

Pulsatile behavior of the medipoapical actomyosin network regulates epithelial elongation changes during morphogenesis (Munjal et al., 2015; Figure 1). Pulsatility of actomyosin network induced by lateral cadherin clusters also promotes dissipation of local tensile stress at the junctions of tightly packed cells limiting apical extrusion of cells out of the epithelia (Wu et al., 2014a,b, 2015; Figure 1). At the medial apical region of cells during intercalation, myosin II contractility initially amplifies then dampens Rho GTPases signaling (Munjal et al., 2015). First, actomyosin contraction locally concentrates (activators of myosin II) Rho and Rho kinase (Figure 1B). Subsequently, this contractility dependent recruitment of actomyosin network and activators then amplifies local RhoGTP activation and tension (Munjal et al., 2015). Second, many F-actin associated regulators, such as formins and coronin 1B, can also organize actomyosin into tensile cortical networks that are highly contractile and stiff (Priya et al., 2016; Acharya et al., 2017). A stiff and tensile actomyosin network would lead to immobilization of the cell cortex (Wu et al., 2014b; Munjal et al., 2015), slowing down the contractility driven recruitment of Rho and Rho kinase. Thus, instead of recruitment, there will be an overall higher rate of dissociation of these actomyosin components. Consequently, relaxation of the actomyosin network occurs (Munjal et al., 2015) (Figure 1B).

Similarly, contractile stress-induced disassembly of tensile actomyosin cables can also relax the actomyosin network (Wu et al., 2014a,b; Jodoin et al., 2015). Actomyosin pulsatility observed at cell-cell junctions (Wu et al., 2014a,b) and medial apical region of cells (Munjal et al., 2015) can be reproduced with computer simulations that model actomyosin as an active fluid, where both extremely dense and low density of simulated actomyosin produces an immobile behavior (Moore et al., 2014). Indeed, overexpression of formins or treatment of cells with jasplakinolide, which increases the density of actomyosin, immobilizes the cortex whereas inhibition of myosin II also leads to an immobile cortex (Munjal et al., 2015; Wu et al., 2014a,b). Only at the intermediate density, cyclical events of contraction and relaxation of a pulsatile network is observed (Moore et al., 2014).

## Myosin II Promotes Stability of Active RhoA at the Zonula Adherens

**FIGURE 2:**
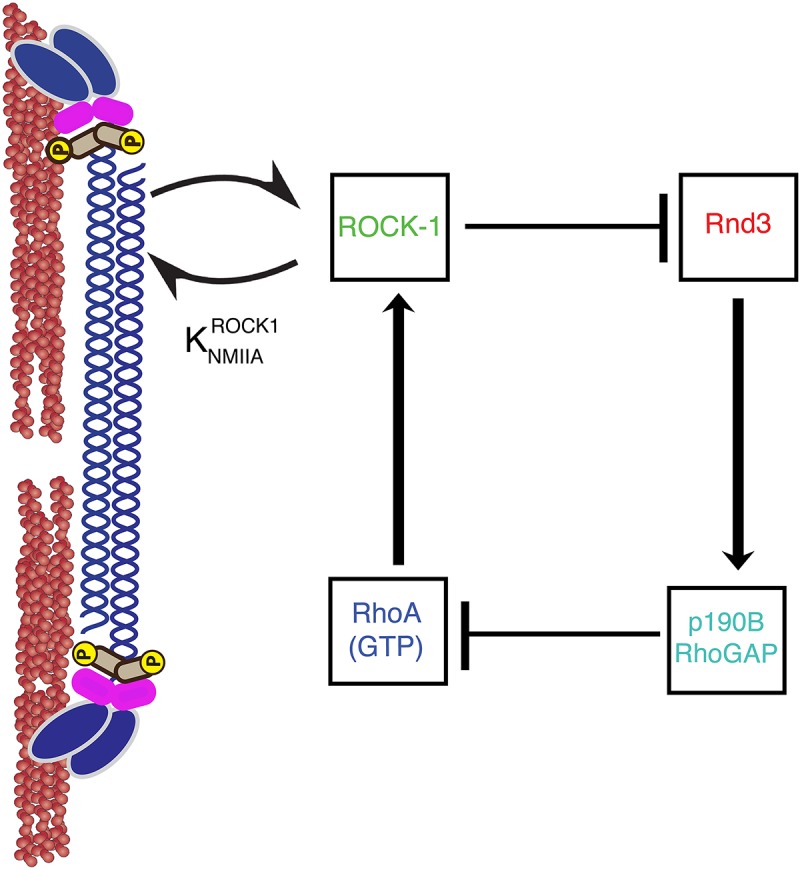
A non-muscle Myosin II minifilament cross linking F-actin filaments (left). ROCK1 binding to myosin II regulates GTP–RhoA, ROCK1, Rnd3, and p190B RhoGAP signaling at the zonula adherens (right). Arrows and T-junctions represent stimulation and repression of junctional recruitment.

In addition, myosin II can scaffold signaling molecules to regulate RhoGTPase activity (Conti and Adelstein, 2008). Myosin II has a head domain that binds and exerts forces on F-actin, and also rod domains which self-associate to form bipolar filaments of Myosin molecules (Figure 2). Strikingly, myosin II can regulate signaling via its rod domain. Indeed, we find that myosin II rod domain plays an important role in preventing the inactivation of RhoA at the zonula adherens. The zonula adherens is an apical cell-cell contact zone of concentrated E-cadherin (Figure 1, 2) which is linked to an enrichment of a variety of cytoskeletal proteins (Smutny et al., 2010; Leerberg et al., 2014; Han et al., 2014). We find that the rod domain of myosin II can further reinforce RhoA GTPases activation by preventing the inactivation of RhoA GTPase (Choi et al., 2008; Smutny et al., 2010; Ma et al., 2012; Priya et al., 2015). How does the rod domain of myosin II prevent the inactivation of RhoA GTPase? This is achieved via myosin IIA rod domain regulation of RhoA GAP signaling (Priya et al., 2015; Figure 2). Rho GTPase is inhibited by GAP and activated by Guanine Exchange Factors (GEFs) (McCormack et al., 2013). In brief, the rod-domain of myosin II scaffolds Rho kinase (ROCK-1) at the zonula adherens, where ROCK-1 phosphorylates Rnd3 GTPase rendering it inactive. Inactivating Rnd3 GTPase is essential to maintain active RhoA because active Rnd3 inhibits RhoA by recruiting and activating p190B Rho GAP (Priya et al., 2015). Thus, myosin IIA recruitment of ROCK-1 supports RhoA signaling by inhibiting the cortical localization of Rnd3-p190B GAP RhoA complex (Priya et al., 2015).

Myosin IIA supports RhoGTPase signaling by scaffolding ROCK-1 independent of myosin IIB. This is consistent with myosin IIA and myosin IIB responding to distinct upstream signals at zonula adherens; myosin IIA is activated by the RhoA pathway while myosin IIB is primarily regulated by Rap1 signaling (Smutny et al., 2010, 2011; Gomez et al., 2015). The rod domain of myosin II exhibits greater dissimilarity in protein sequence between different paralogs and thus, could potentially explain why these myosin isoforms respond to distinct signaling pathways (Conti and Adelstein, 2008; Vicente-Manzanares et al., 2009; Heissler and Manstein, 2013). For example, the rod domain of myosin II harbors unique regulatory modifications like phosphorylation sites (Rosenberg and Ravid, 2006).

## Myosin II Regulates Rac and CDC42 GTPases

Myosin II can also affect Rac GTPase signaling. The GEFs that activates Rac-1 includes Tiam-1 and β-pix. GEFs including Tiam-1 and β-pix activates Rac-1 by binding to the inactive Rac-1 GDP, catalyzing the sequential release of GDP and binding of GTP, to activate Rac-1. Rac GTPase signaling regulates cell migration, by stimulating cellular protrusions through inducing Arp2/3 branched actin formation (Ridley, 2015). Interestingly, consistent with the commonly reported antagonism between Rac1 and RhoA, myosin II can activate RhoA but dampen Rac activation. Indeed, myosin IIA was implicated as negative regulator of Rac GTP dependent cell migration (Even-Ram et al., 2007). Inhibition of myosin IIA stabilized microtubules which then recruited Rac GEF Tiam1 to activate Rac GTPase at the leading edge of cells. Thus, active myosin II can retard cell migration by reducing Tiam-1 mediated Rac activation. Alternatively, active myosin II can also dampen Rac GTPase signaling by sequestering Rac1-GEF β-pix from RacGTPase (Kuo et al., 2011). Inhibiting myosin II by blebbistatin or plating fibroblasts on a compliant substrate to reduce cellular contractility stimulated the localization of Rac1-GEF β-pix to focal-adhesions thus activating Rac-1. Similarly, the gain of function experiments in CHO epithelial cells, by expressing myosin II phosphomimetic regulatory light chain (RLD-DD) reduces the localization of Rac GEFs β-pix to cortical Rac GTPase, thus dampening Rac GTPase activation (Vicente-Manzanares et al., 2011). Taken together, myosin II can retard cell-migration by preventing the colocalization of Rac1 activators such as Tiam-1 and β-pix to Rac GTPase.

Additionally, myosin II is reported to affect the Rho GTPase-CDC42. Myosin II was found to regulate neuronal morphology by modulating CDC42 signaling (Shin et al., 2014). In the growth cone of hippocampal cells, blebbistatin mediated inhibition of myosin II released the β-pix GEFs from myosin II-GEF complex to associate with and activate CDC42. Thus, promoting actin dependent protrusions and filopodia formation from the neurite shaft.

Then, how does myosin II block the recruitment of these activators to Rac and CDC42? Interestingly, myosin II was found to directly interact with various Dbl family of GEFs including β-pix and Tiam1 (Lee et al., 2010). The ATPase activity and actomyosin filament assembly were necessary for this interaction. Indeed, blebbistatin, ATPase-defective mutants or obliteration of filamentous-actin from the lysates compromised myosin II interaction with these GEFs. Myosin II perturbs the catalytic activity of these bound GEFs. Thus, the contractile actomyosin filament may suppress cell-migration by sequestering Dbl-family of GEFs which activates Rac and CDC42.

In summary, myosin II regulates Rho GTPase signaling in many developmental related processes including cell adhesion, migration and morphogenesis. Since Rho GTPases signaling are commonly misregulated in cancer (Lozano et al., 2003), how myosin II-regulated signaling is regulated in cellular processes related to cancer including cell-contact inhibition of growth (Chiasson-MacKenzie et al., 2015) and epithelial-to-mesenchymal transition (Thiery and Sleeman, 2006; Mangold et al., 2011; Greenlees et al., 2015; Wu et al., 2015) remains to be explored.

## Author Contributions

SW and RP wrote the manuscript and made figures.

## Conflict of Interest Statement

The authors declare that the research was conducted in the absence of any commercial or financial relationships that could be construed as a potential conflict of interest.
